# Primary Cutaneous Gamma-Delta T-Cell Lymphoma Initially Diagnosed as Subcutaneous Panniculitis-like T-Cell Lymphoma with Dermatomyositis

**DOI:** 10.3390/dermatopathology9020018

**Published:** 2022-04-29

**Authors:** Chika Hirata, Kozo Nakai, Yusuke Kurasawa, Naoki Maekawa, Shuichi Kuniyuki, Keiko Yamagami, Masahiko Ohsawa, Daisuke Tsuruta

**Affiliations:** 1 Department of Dermatology, Osaka City University Graduate School of Medicine, Osaka 545-8585, Japan; nakai.kozo@med.osaka-cu.ac.jp (K.N.); dtsuruta@med.osaka-cu.ac.jp (D.T.); 2 Department of Dermatology, Osaka City General Hospital, Osaka 534-0021, Japan; kuranosuke0020@yahoo.co.jp (Y.K.); heipme4731@gmail.com (N.M.); ga28547@wa2.so-net.ne.jp (S.K.); 3 Department of Internal Medicine, Osaka City General Hospital, Osaka 534-0021, Japan; yamasanz@qb3.so-net.ne.jp; 4 Department of Diagnostic Pathology, Osaka City University Graduate School of Medicine, Osaka 545-8585, Japan; m1311435@med.osaka-cu.ac.jp

**Keywords:** primary cutaneous gamma-delta T-cell lymphoma, WHO-EORTC classification, dermatomyositis

## Abstract

Primary cutaneous gamma-delta T-cell lymphoma (CGD-TCL) is a rare cutaneous lymphoma. Panniculitis-like T-cell lymphoma (SPTCL) has a better prognosis than CGD-TCL. SPTCL is sometimes associated with autoimmune disease. A 64-year-old Japanese female with a history of dermatomyositis presented with subcutaneous nodules on the upper extremities and exacerbated dermatomyositis. A skin biopsy showed lobular panniculitis, a vacuolar interface change, and a dermal mucin deposit. Fat cells rimmed by neoplastic cells, fat necrosis, and karyorrhexis were observed. The atypical lymphoid cells showed CD3+, CD4−, CD8+, granzyme B+, CD20−, and CD56−. Polymerase chain reaction analysis demonstrated a T-cell receptor rearrangement. The patient was initially diagnosed with SPTCL, so the dose of prednisone was raised from 7.5 to 50 mg daily (1 mg/kg). After one month, erythematous nodules regressed, and muscle symptoms improved. Subsequently, prednisone was tapered, and cyclosporin A was added. After one year, the patient remained symptom-free and continued taking 7.5 mg prednisone and 100 mg cyclosporin A daily. Afterward, we immunostained skin samples with antibodies against TCR-ß and δ and found positive TCR-δ and negative TCR-ß. Therefore, we corrected the diagnosis to CGD-TCL, although the clinical course and the presence of dermatomyositis were reminiscent of SPTCL.

## 1. Introduction

Primary cutaneous gamma-delta T-cell lymphoma (CGD-TCL) is defined as a cytotoxic T-cell lymphoma with clonal proliferation of mature, activated gamma-delta T-cells [1]. Previously, CGD-TCL was classified as a subcutaneous panniculitis-like T-cell lymphoma (SPTCL), characterized as an SPTCL with gamma-delta T cells [2]. In the latest WHO-EORTC classification, SPTCL was defined as a cytotoxic alpha-beta T-cell lymphoma characterized by the primary involvement of subcutaneous tissue. Consequently, cytotoxic T-cell lymphoma with a gamma-delta phenotype was classified as a primary cutaneous peripheral T-cell lymphoma and called CGD-TCL [3]. This separation was due to the favorable prognosis of SPTCL, its CD4−, CD8+, and CD56− phenotype, and its occasional association with autoimmune diseases. In contrast, CGD-TCL has a poor prognosis (11% 5-year overall survival) and a CD4−, CD8−, and CD56+ phenotype [3]. Here, we describe a case of CGD-TCL that presented with a CD4−, CD8+, and CD56− phenotype associated with dermatomyositis and an apparent indolent course, which we initially diagnosed as SPTCL.

## 2. Case Report

**Figure 1:**
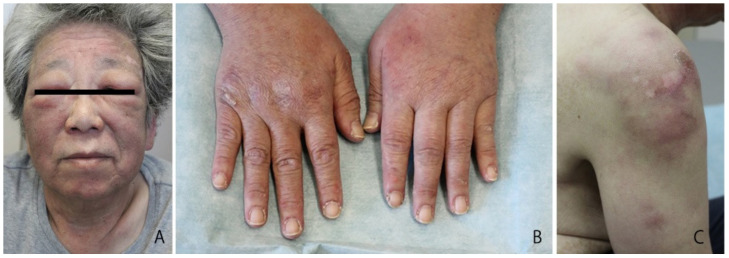
Presentation of the patient. (**A**) Facial edema and edematous eyelids; (**B**) scaly erythema on the dorsal surface of the digits; (**C**) subcutaneous nodules on the brachium.

**Figure 2:**
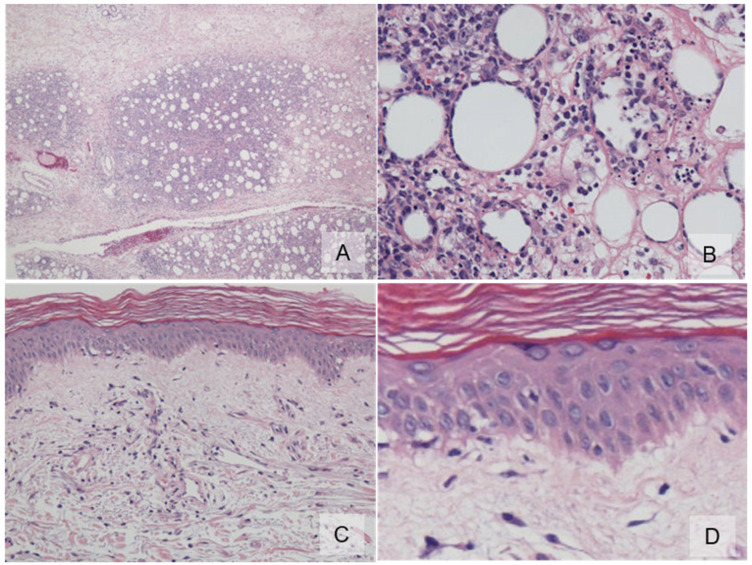
Histopathology. (**A**) An inflammatory infiltrate was found exclusively in the subcutis with a lobular pattern (×100); (**B**) subcutis lobule displays individual fat cells rimmed by neoplastic mononuclear cells, fat necrosis, and karyorrhexis (×400); (**C**) the overlying epidermis showed vacuolar interface changes (×100); (**D**) high-power view of basal vacuolar change (×400).

**Figure 3:**
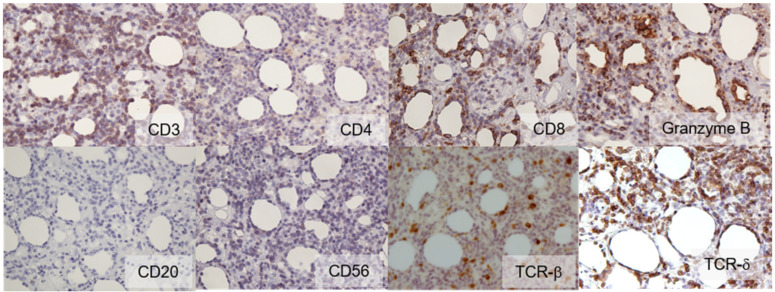
Immunohistochemistry (×400). Atypical lymphoid cells showed the following expression profile: CD3 (+), CD4 (−), CD8 (+), granzyme B (+), CD20 (−), CD56 (−), TCR-β (−), and TCR-δ (+).

In 2011, a 64-year-old Japanese female with a known case of dermatomyositis for a year presented with typical skin rash, myositis, and interstitial pneumonia. No malignancy was reported before the visit. She had no fever, no night sweats, and no weight loss. She had been treated with oral prednisone 7.5 mg daily. Two months prior to visiting our hospital, she had noticed painful, indurated nodules on the upper arm, and she presented with general fatigue and proximal muscle weakness. An examination revealed no fever, but facial edema, edematous eyelids (Figure 1A), scaly erythema on the dorsal surfaces of her digits (Figure 1B), subcutaneous nodules on the upper extremities (Figure 1C), and muscle strength grade 3 of 5 in the proximal upper and proximal lower limbs. Laboratory investigations showed a normal white blood cell count and mild anemia. Although her creatine kinase levels were normal, she had high levels of lactate dehydrogenase (407 IU/L) and soluble interleukin-2 receptor (2660 U/mL). A clinical evaluation of a serum sample revealed antinuclear antibodies (ANA; 1:320) with a speckled pattern. Serum studies produced negative results for Sjogren’s syndrome antibodies (SS-A/Ro, SS-B/La) and Jo-1 syndrome antibodies (anti-Jo-1). Computed tomography of the chest, abdomen, and pelvis showed interstitial changes in the lung without lymphadenopathy. Magnetic resonance imaging of the upper arm showed high signal intensities in the biceps brachii muscle and subcutaneous fat tissue on a short tau inversion recovery sequence. A muscle biopsy from the right biceps brachii muscle revealed perivascular lymphocytic inflammation and muscle fiber atrophy. A skin biopsy showed lobular panniculitis (Figure 2A). In addition, we observed individual fat cells rimmed with neoplastic mononuclear cells, fat necrosis, and karyorrhexis (Figure 2B). The overlying epidermis showed vacuolar interface changes (Figure 2C,D). We identified atypical lymphoid cells. The atypical lymphoid cells were positive for CD3, CD8, and granzyme-B, but they did not mark with CD20, CD4, and CD56 (Figure 3). The result of EBV-encoded RNA in situ hybridization was negative. A polymerase chain reaction analysis demonstrated a T-cell receptor (Jγ and Cβ) monoclonal rearrangement. In addition, we observed vacuolar interface changes and dermal mucin deposits. Based on the 2008 WHO classification, the patient was diagnosed with SPTCL and exacerbated dermatomyositis. For treatment, the prednisone dose was raised from 7.5 to 50 mg/day (1 mg/kg per day). After one month, the erythematous nodules of the upper limbs regressed, and muscle symptoms improved. Subsequently, prednisone was tapered to 7.5 mg/day, and cyclosporin A (100 mg/day) was added. This treatment did not exacerbate the symptoms, and after 1 year, the patient remained symptom-free and continued taking prednisone (7.5 mg/day) and cyclosporin A (100 mg/day). The patient was subsequently transferred; thus, her later course is unknown.

In 2020, we performed immunohistochemistry on skin samples. We stained samples with antibodies against TCR-ß and TCR-δ. We observed positive results for TCR-δ expression and negative results for TCR-ß expression (Figure 3). Based on the 2018 WHO classification, we corrected the initial diagnosis of SPTCL to a diagnosis of CGD-TCL.

## 3. Discussion

CGD-TCL is defined as a cytotoxic T-cell lymphoma with the presence of gamma-delta T cell receptor (TCR) expression and the absence of alpha-beta TCR expression [1]. T cells can be divided into alpha-beta-TCR T cells and gamma-delta-TCR T cells based on their TCR expression. The vast majority of circulating and peripheral T cells express the alpha-beta TCR, which is involved in the adaptive immune response. However, some T cells express the gamma-delta TCR, which is thought to be involved with the innate immune system at the epidermal and mucosal surfaces; thus, they are typically confined to the skin, mucosa, and submucosa of the gastrointestinal tract [4]. A condition similar to CGD-TCL presents primarily in mucosal sites, but it remains unclear whether cutaneous and mucosal gamma-delta T-cell lymphomas are the same disease [2]. Consequently, CGD-TCL is categorized as a primary cutaneous peripheral T-cell lymphoma, and this category is considered to include a heterogeneous group of cutaneous T-cell lymphomas [2].

SPTCLs are negative for gamma-delta TCR [5]. SPTCLs generally show CD4−, CD8+, and CD56− expression; they generally have a good prognosis; and they are sometimes associated with autoimmune diseases. Moreover, they can be effectively treated with an immunosuppressive regimen. In contrast, CGD-TCL typically shows CD4−, CD8− (CD8+ in rare cases), and CD56+ expression, and it is an aggressive disease that is resistant to multiagent chemotherapy [1]. In our patient, at the time of diagnosis, delta TCR was only detectable in frozen tissues; therefore, we could not perform an immunohistochemical analysis for the delta TCR. Consequently, our initial diagnosis was SPTCL, based on clinicopathological and prognostic features. Later, after the advancement of immunohistochemical techniques, we corrected the diagnosis to CGD-TCL. 

Although CGD-TCL typically has an aggressive course and a poor prognosis, with a median survival of approximately 12 months [1], indolent cases of CGD-TCL have also been reported [6,7,8,9]. Indolent CGD-TCL cases displayed recurrent self-remitting nodules, which mimicked lupus panniculitis or erythema nodosum, and lasted for several years. Similar to our case, those cases responded very well to treatment with either multiagent chemotherapy or prednisone [6,7]. Although some patients with indolent courses survived disease-free after treatment, others rapidly progressed and had unfortunate outcomes [8,9]. In our patient, the disease appeared to be indolent, but the prognosis will not be clear for several years. 

CGD-TCL is rare; it accounts for less than 1% of primary cutaneous lymphomas [1]. Consequently, the relationship between CGD-TCL and autoimmune diseases remains unclear. A few previous cases were reported to resemble lupus profundus [6,9,10], systemic vasculitis [10], and dermatomyositis [10]. A study suggested that the highest risk for malignancy is seen in the first year after dermatomyositis diagnosis [11]. It is also reported that the prevalence of non-Hodgkin lymphoma is highest in hematopoietic malignancies [11]. However, the association between dermatomyositis and malignancies during periods of exacerbation is unknown. In the present case study, we observed a heliotrope rash, shawl and V signs, symmetric muscular weakness, myalgia, and positive ANA. These signs led to a diagnosis of dermatomyositis, but the diagnosis could not be confirmed due to the absence of muscular inflammation in a histopathological examination [10].

Although typical CGD-TCL has a poor prognosis, and SPTCL is typically associated with autoimmune diseases, the present study revealed that CGD-TCL is a heterogeneous disease with a variable prognosis, and it is sometimes associated with an autoimmune disease. More CGD-TCL case studies will help to elucidate this rare disease further.
